# Production of natural flavor compounds using *Bacillus subtilis-*fermented soybean meal extract and their biological potential: a comprehensive *in vitro* study

**DOI:** 10.3389/fnut.2023.1280209

**Published:** 2024-01-16

**Authors:** Abeer M. Abd-Alrahman, Manal M. Ramadan, Mohamed F. Maraay, Rabab Salem, Fatma M. Saleh, Mahmood A Hashim, Anastasia Zhernyakova, Tamer M. El-Messery

**Affiliations:** ^1^Department Food Science and Technology, Faculty of Home Economics, Al-Azhar University, Tanta, Egypt; ^2^Department Chemistry of Flavor and Aroma, Food Industry and Nutrition, National Research Centre, Cairo, Egypt; ^3^Microbial Chemistry, National Research Center, Cairo, Egypt; ^4^International Research Centre “Biotechnologies of the Third Millennium,” Faculty of Biotechnologies (BioTech), ITMO University, Saint Petersburg, Russia; ^5^Agricultural Research Center, Food Technology Research Institute, Giza, Egypt; ^6^Department of Food and Nutrition, Faculty of Agriculture and Forestry, University of Helsinki, Helsinki, Finland

**Keywords:** soybean meal, solid-state fermentation, *Bacillus subtilis*, L-Lysine, L-Threonine and biological effects

## Abstract

This study aims to investigate the production of natural flavor compounds through the utilization of *Bacillus subtilis*-fermented soybean meal extract and evaluate their biological potential. The experiment involved a comprehensive *in vitro* investigation to assess the capabilities and effects of the produced flavor compounds. The resulting flavor compounds were subjected to various *in vitro* tests to assess their properties, including cytotoxicity, antioxidant activity, anticancer potential, antiviral activity, and antimicrobial activity. To enhance the fermentation process, soybean meal extract was fortified with a combination of L-Lysine and L-Threonine. Gas chromatography-mass spectrometry (GC/MS) analysis was conducted on the fermented soybean meal using two strains of *Bacillus subtilis*, namely *NRCH123* and *NRCZ144*. This analysis revealed the presence of various volatile compounds in all extracts, including Butylated hydroxytoluene. The fermented soybean extract with *bacillus subtilis* NRCZ144 (B2) fortified with a combination of 2.5% (w/w) L-Lysine and 2.5% w/w L-threonine (SLT2) exhibited a rich profile of flavor compounds, with Eucalyptol being identified as the predominant compound. The antioxidant activity of the SLT_2_ extract was found to be 72.04% at a concentration of 100 μg/mL, indicating significant antioxidant potential. Furthermore, when tested against the human liver cancer cell line HepG2, the extract demonstrated anticancer activity with an IC50 value of 2.26 μg/mL. The extract exhibited potent cytotoxicity, with an IC50 value of 1.02 μg/mL. Importantly, the SLT_2_ extract displayed strong antibacterial and antifungal activity, even at very low concentrations. The extract’s antimicrobial properties indicate its potential for inhibiting the growth of bacteria and fungi.

## Introduction

Flavor plays a crucial role in food, significantly impacting the sensory characteristics and overall appeal of food products to consumers. Food flavor is a crucial characteristic that encompasses various sensory aspects, including taste, smell, texture, temperature, and pungency. The combination of odor and taste plays a significant role in determining the overall flavor profile and its discernment by individuals (1). Flavor is a fundamental component of food that greatly influences the organoleptic properties and consumer acceptance of food products (2).

The flavor of food can be modified using natural or artificial flavorings (3). Artificial flavors are chemically synthesized and do not occur naturally (4). On the other hand, natural flavors are derived from natural sources and do not contain artificial ingredients. They can be obtained from various natural substances such as spices, fruit juices, vegetables, herbs, and animal-derived products like meats, poultry, seafood, eggs, and dairy (5, 6).

The production of artificial flavors has been associated with environmentally unfriendly processes and the generation of unwanted byproducts, which can reduce process efficiency and pose health risks (7, 8).

Natural flavor compounds have shown potential positive effects in the prevention of various diseases, including cancer and neurological disorders like Alzheimer’s disease. These benefits are often attributed to the presence of phenolic compounds, which possess properties such as free radical scavenging, anti-inflammatory activity, antiviral effects, and antimicrobial properties. These compounds not only contribute to disease prevention mechanisms but also enhance the stability of food products by exerting antimicrobial effects (9, 10).

Research and development of novel natural flavors are of great importance in modern society (11, 12). Natural flavor agents (NFAs) are produced through extraction from natural materials or by utilizing microbial fermentation or enzymatic processes to convert natural precursors into desired flavors (13).

Fermentation methods, including submerged fermentation (SmF) and solid-state fermentation (SSF), are used for the production of NFAs. SSF has gained significant attention as a promising method due to its advantages such as higher yields, lower energy requirements, and utilization of agricultural residues and waste materials (14). Agricultural residues are rich in bioactive compounds and have been extensively utilized as raw materials in various research and industrial applications (15). These agro-wastes are attractive for biotechnological flavor production through microbial fermentation due to their high content of reusable components for microorganisms (16).

Soybean meal, the residual material obtained after soybean oil extraction, serves as a suitable substrate for the growth of microorganisms, particularly certain Bacillus species like *B. subtilis* (17, 18).

This study aims to develop a novel approach to flavor compound production by utilizing *Bacillus subtilis* fermentation of soybean meal as a secondary product. The comprehensive evaluation of the extract’s biological properties, along with the incorporation of amino acid fortification and the identification of flavor compounds, contributes to advancing knowledge in the field of natural flavor compounds and their potential applications. Additionally, the anticancer, antioxidant, antimicrobial, and antiviral activities of these flavors is evaluated, along with an assessment of their cytotoxicity.

## Materials and methods

### Raw materials

Soybean meal (48.90% Protein, 35.80% Carbohydrate, 5.30% Ash and 1.00% Fat) and Sugar beet pulp (65.40% Carbohydrate, 9.20% Protein, 7.20% Ash and 0.90% Fat) were purchased from Food Technology Research Institute, Agric. Res. Center, Giza, Egypt.

### Microorganisms

Tow strains of Bacilli bacterial strains belonging to *Bacillus subtilis*, namely *Bacillus subtilis NRCH123* (B1), *Bacillus subtilis NRCZ144* (B2) were purchased from Microbial Chemistry Lab., National Research Centre, Dokki, Giza, Egypt.

### Chemicals and reagents

Amino acid (L-Lysine and L-Threonine), yeast extract, diethyl ether, and sodium sulphate anhydrous were purchased from Sigma Aldrich Chemical Co. (St. Louis, MO, USA).

## Technical methods

### Biotechnology to produce natural compounds

#### Inoculum preparation

*Bacillus subtilis* strains B1 and B2 were cultured on nutrient agar medium in petri dishes and incubated at 27°C for 4 h. To recover the cells, each petri dish was soaked with 5 ml of sterile distilled water, using the method described by Besson et al. (19).

#### Solid state fermentation with soybean meal

The fermentation process was conducted using solid-state fermentation in 250 ml conical flasks for both strains B1 and B2. Each strain consisted of five samples, including a control sample without any added nutrients Table 1. The fermentation process was carried out as follows:

*1. Preparation of soybean meal substrate:*

– Five grams of soybean meal were moistened with distilled water at a ratio of 2:1 (w/v).
– The soybean meal substrate was then autoclaved at 121°C for 15 min to ensure sterilization.

*2. Inoculation and incubation:*

– One milliliter of bacterial strain B1 or B2 was added to each flask containing the sterilized soybean meal substrate.
– The flasks were then incubated at 27°C for 72 h to start fermentation.

*3. Nutrient supplementation:*

– For the control sample, no additional nutrients were added.
– The remaining four samples received different nutrient supplements, which were added before the sterilization step.
– The first sample received 2.5% (w/w) L-Lysine and 2.5% (w/w) L-Threonine.
– The second sample received 5% (w/w) L-Lysine.
– The third sample received 5% (w/w) L-Threonine.
– The fourth sample received 5% (w/w) yeast extract.

**TABLE 1:** Symbols samples of cultivation medium.

Microorganisms	Soybean meal (g)	Nutrient (w/w %)			Symbol
		L-Lysine	L-Threonine	Yeast extract	
B_1_	5	–	–	–	Con_1_
B_1_	5	2.5	2.5	–	SLT_1_
B_1_	5	5	–	–	SL_1_
B_1_	5	–	5	–	ST_1_
B_1_	5	–	–	5	SY_1_
B_2_	5	–	–	–	Con_2_
B_2_	5	2.5	2.5	–	SLT_2_
B_2_	5	5	–	–	SL_2_
B_2_	5	–	5	–	ST_2_
B_2_	5	–	–	5	SY_2_

### Volatile compounds isolation

The volatile compounds present in each sample were extracted using a dynamic headspace system. Approximately 20 g of each sample was placed in a conical flask along with 250 ml of distilled water. The mixture was then stirred at 60°C using a Teflon-coated magnetic bar (B48.5). Nitrogen gas was purged through the samples for a duration of 3 h at a flow rate of 100 ml/min. The volatile compounds in the headspace were carried into cold traps containing diethyl ether and maintained at a temperature of −10°C. The solvents containing the volatiles were subsequently dried using anhydrous sodium sulfate. The solvents containing the volatile matter were concentrated to a final volume of 100 μl using a rotary evaporator (Heidolph, HEID31011, Germany) under normal pressure at 40°C, following the procedure outlined by Abd El-Mageed (20). The resulting extracts were then stored at −20°C until further experimentation.

### Analytical methods

#### Gas chromatographic-mass spectrometric (GC/ MS) analysis

The GC-MS system (Agilent Technologies) was equipped with gas chromatograph (7890B) and mass spectrometer detector (5977A) at Central Laboratories Network, National Research Centre, Cairo, Egypt. Samples were diluted with hexane (1:19, v/v). The GC was equipped with HP-5MS column (30 m × 0.25 mm internal diameter and 0.25 μm film thickness). Analyses were carried out using hydrogen as the carrier gas at a flow rate of 1.0 ml/min at a split 1:20 of, injection volume of 1 μl and the following temperature program: 40°C for 1 min; rising at 4°C /min to 150°C and held for 6 min; rising at 4°C/min to 210°C and held for 1 min. The injector and detector were held at 280°C. Mass spectra were obtained by electron ionization (EI) at 70 eV; using a spectral range of m/z 50–550 and solvent delay 3 min. Identification of different constituents was determined by comparing the spectrum fragmentation pattern with those stored in Wiley and NIST Mass Spectral Library data. The retention indices (Kovats index) of the separated volatile compounds were calculated with reference to the retention time of a series of n-alkanes (C8–C22) as external standard, run at the same conditions. The isolated peaks were identified by matching them with data from the library of mass spectra (National Institute of Standard and Technology, NIST) and comparing with those of authentic compounds and published data (21). The percentage composition of each sample was computed by the normalization method from the GC peak area, calculated by mean of three injections.

#### Cytotoxicity assay of SLT_2_ extract

The MTT assay was utilized. This method is a sensitive, quantitative, and reliable colorimetric technique that measures cell viability. The absorbance of the resulting colored solution is quantified using a spectrophotometer, typically at a wavelength between 500 and 600 nm (22). The cytotoxic effect of the extracts was evaluated on African green monkey (Vero) cell lines obtained from the American Type Culture Collection (ATCC-clone CCL-81). The cytotoxicity of each extract was expressed as the 50% cytotoxic concentration (IC50), which represents the concentration required to reduce cell viability to 50% of the control.

#### Anticancer activity of SLT_2_ extract

In assessing the anticancer activity of the SLT_2_ extract, the cytotoxic effect on human liver cancer cells (HepG2) was measured. The cell viability was determined using the MTT assay, and the percentage of viability was calculated as follows: Cell viability percentage = (OD of treated cells / OD of untreated cells) × 100 (23).

#### Antiviral activity of SLT_2_ extract

**a. Propagation of test virus on Vero cells**

Hepatitis A virus (*HAV*) (Applied Research Sector VACSERA -Egypt) was propagated on Vero cells using the method described by Afshar (24) and Joseph and Thomas (25).

**b. Evaluation of antiviral activity against test virus**

Antiviral activity of extracts safe concentrations against Hepatitis A *(HAV)* virus was determined according to Petricevich and Mendonça (26), where the virus was titrated on cell lines post 24 h treatment with and without the extract. The difference between the virus titers in the untreated and treated cells represents the antiviral activity.

**c. Evaluation of antiviral activity in cells pretreated with the extract**

Vero cells were pretreated with extracts for 24 h followed by viral inoculation to evaluate the effect of the initial stages of viral replicate, as in Steven et al. (27). The 50 % endpoint was calculated according to Sberna et al. (28) as follows:

50%endpoint(CCID)50=%CPE>50-50ConstXlogdilution%CPE>50%-%CPE<50%

#### Antioxidant activity of SLT_2_ extract

The radical scavenging activity was assessed using the DPPH• method, as described by Guleria et al. (29). To perform the assay, the extract (natural flavor compounds) at different concentrations (25, 50, and 100 μg/ml) was mixed with 1 ml of a 90 μM DPPH• solution in methanol. The total volume was adjusted to 4 mL using methanol. The mixtures were thoroughly mixed and incubated at 25°C in the dark for 1 h. After incubation, the absorbance of the solutions was measured at 517 nm.

The radical scavenging activity (RSA) was calculated as the percentage of DPPH• discoloration using the following equation:

%⁢Antioxidan⁢activity=[(A⁢0-A⁢s)⁢A⁢0]×100,

where A0 represents the absorbance of the control (containing all reagents except the test compound), and As represents the absorbance of the test compound. The inhibitory concentration at which 50% inhibition (IC50) occurred was determined by plotting the percentage of inhibition against the concentration of the extract. BHT was used as a reference compound in this assay.

#### Antimicrobial activity of SLT_2_ extract

**a. Microorganisms:**

The inhibitory effect of the sample extract was assessed against two strains of foodborne pathogenic bacteria: *Staphylococcus aureus* (Gram-positive) and *Escherichia coli* (Gram-negative). The stock cultures of these bacteria were grown on nutrient agar slants at 37°C for 24 h and then stored in the refrigerator until further use.

For the antifungal assay, two fungal species were employed: *Aspergillus niger* and *Candida albicans*. The stock cultures of these fungi were grown on potato dextrose agar slants at 25°C for 5 days and similarly stored in the refrigerator until use.

**b. Disc diffusion technique:**

The sensitivity test of the sample extract was determined using the disc diffusion method, following the Kirby-Bauer technique (30). Inhibition zones were measured and expressed as the diameter of the clear zone, including the diameter of the paper disc (31). The antifungal activity was evaluated by measuring the zone of inhibition (in millimeters) against the tested fungus (32).

**c. Determination of minimum inhibitory concentration (MIC):**

The minimum inhibitory concentration (MIC) for the SLT_2_ extract was determined using the microbroth dilution method. The MIC against fungi was determined following the technique described by Marrez and Sultan (33).

#### Sensory evaluation of SLT_2_ extract

The evaluation was conducted by a well-trained panel consisting of 20 members (ten female and ten male) drawn from the National Research Center, Cairo, Egypt, according to Fadel et al. (34) with some modifications. Sensory analysis was carried out to determine the odor sensory attributes of the sample to estimate the odor acceptability. The odor acceptability was assessed by a five point scale (1: highly unacceptable, 2: unacceptable, 3: average, 4: acceptable, 5: highly acceptable).

#### Statistical analysis

All treatments were done three times, and the results were computed as the average standard deviation of the three trials (SD).

## Results and discussion

### Gas chromatographic-mass spectrometric (GC/ MS) analysis

#### GC/MS analysis of the Con_1_ extract

**FIGURE 1:**
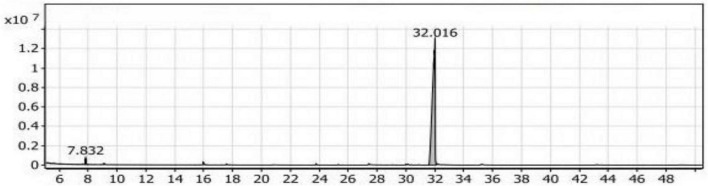
Gas chromatography-mass spectrometry (GC/MS) chart of soybean meal fermented by *Bacillus subtilis NRCH123* using solid-state fermentation.

The volatile compounds in soybean meal fermented by *Bacillus subtilis NRCH123* without bacterial stimuli were extracted and analyzed using GC/MS, as shown in Table 2. This analysis aimed to determine whether the strain *Bacillus subtilis NRCH123* has the ability to produce flavor compounds. The results indicated the identification of two volatile compounds, each belonging to a different chemical group. The first compound, 2,3-dimethyl-heptane, belongs to the group of aliphatic compounds and represents the lowest percentage (1.08%). The second group is phenols, and the compound Butylated hydroxytoluene (BHT) represents this group, with a percentage of 98.92% and a retention time of 32.016 (Figure 1).

**TABLE 2:** Gas chromatography-mass spectrometry (GC/MS) analysis of soybean meal fortified with or without L-Lysine + L-Threonine fermented by *Bacillus subtilis NRCH123* and *NRCZ144* using solid-state fermentation.

Type	Identified compounds	Retention indices	*Con*_1_	*SLT*_1_	*SL*_1_	*ST*_1_	*SY*_1_	*Con*_2_	*SLT*_2_	*SL*_2_	*ST*_2_	*SY*_2_
Aliphatic compounds	3-ethyl-3-methyl-Pentane	744.17			18.35	19.60	13.42			19.89	18.12	25.40
	Di-sec-butyl ether	763.68			16.46	19.50	13.87			17.59	12.19	18.85
	1-(1-methylpropoxy)-Butane	766.29									7.18	
	3-methyl-Heptane	774.64					4.81					
	2,4-dimethyl-heptane	786.14		1.37								
	2-Ethoxypentane	806.60				1.34	1.97			1.31		
	Hydroperoxide, 1-methylhexyl	813.26										1.30
	2,3-dimethyl-heptane	816.41	1.08								1.91	
	3-ethyl-3-methyl-Hexane	816.45				0.66	1.36					
	2,2-dimethyl-1,1-difluorocyclopropane	829.80				0.44						
	P-Xylene	863.07									1.23	
	1,2-dimethyl-Benzene	863.96			1.11					1.15	1.42	
	2-butoxy-Pentane	878.84					0.27					
	3-Ethyl-3-methylheptane	927.49			2.34	2.74	3.32			2.67	2.45	2.33
	Eucalyptol	1029.27							31.25			1.87
	3-ethyl-2,5-dimethyl-Pyrazine	1089.66							26.14			
	4,5-diethyl-Octane	1135.52			0.53	0.65	0.60			0.35	0.24	0.51
	5,6-dimethyl-Decane	1153.55				0.24	0.20			0.26		0.33
	2,3,5,8-tetramethyl-Decane	1194.94				0.37	0.36			0.41	0.33	0.38
	delta.-Elemene	1384.41							0.54			
	Copaene	1425.45							1.20			
	Caryophyllene	1470.76							8.25			
	*Trans-*caryophyllene	1471.15		1.00								
	Butylated hydroxytoluene	1501.03	98.92	96.40	0.76	0.76	6.52	100	15.00		4.25	3.76
	2-(phenylmethyl)-1,3-Dioxolane	1714.63				0.26						
	Oxirane, 2-[2-(benzyloxy)-1-(1-methoxy-1-methylethoxy)ethyl]	1715.36			0.28					0.30		
Phenols	4,6-di-tert-Butyl-m-cresol	1513.85			0.91	0.56	0.48			0.98	0.84	0.34
	2,6-bis (1,1-dimethylethyl)-4-methyl-Phenol	1893.49					0.30			0.54		
Alcohols	3-methyl-2-Hexanol	737.87					1.19					0.43
	Trimethylsilylmethanol	799.72			19.70	18.56	15.56			19.21	18.64	17.21
	1-propoxy-2-Propanol	801.95			0.93		0.55			1.16	1.97	0.92
	3-(1-ethoxyethoxy)- 2-Methylbutane-1,4-diol	809.57			1.29							
	2,3-Butanediol	823.30					2.99					
	2-ethenyl-Bicyclo[2.1.1]hexan-2-ol	835.92			4.33							
	5-methyl-2-Hexanol	837.26				1.88	1.39				2.09	
	2,4-dimethyl-3-Pentanol	871.77			1.88	2.66	2.99			2.7	2.14	2.32
	3,5-dimethyl-3-hexanol	916.92			0.64	0.62	0.57			0.38	1.08	0.30
	3-Allyloxy-1,2 propanediol	924.54			1.44	1.31	0.96				1.20	1.21
le	4,4-dimethyl-2-Pentanol	935.11			1.74		0.20			1.88	1.95	
	3-(1,3-dimethylbutoxy)-2-Butanol	963.35			1.15	1.04	1.19			1.15	1.14	
	2,2-dimethyl-1-Octanol	999.19			1.41	1.05	1.03			0.96	1.46	1.31
	2,4-dimethyl-3-Heptanol	1035.40			1.19	2.01	1.60			1.34		
	1,6-Dideoxy-l-mannitol	1056.20			0.30	0.26	0.20			0.35	0.24	
	2,6-dimethyl-7-Octen-2-ol	1082.77			0.34	0.25	0.27			0.32	0.28	0.35
	3-(1,3-dimethylbutoxy)-2-Butanol	1130.66			0.27	0.21	0.25				0.22	
e	3,4-dimethyl-2-Hexanol	1157.05			0.28	0.24				0.34		1.31
	3-Ethyl-3-methyl-2-pentanol	1164.86				0.34	0.26					
	2-butyl-1-Octanol	1194.94			0.41		0.31					
	exo-2,7,7-trimethylbicyclo[2.2.1]heptan-2-ol	1226.328			0.29	0.22					0.26	
	Terpineol	1225.40							2.55			
	2,2-dimethyl-1-Pentanol	1261.25			0.20	0.89	0.19			0.22		
	E-11,13-Tetradecadien-1-ol	1436.76		1.23								
	2,6-bis (1,1-dimethylethyl)-1,4-Benzenediol	2002.88				0.20	0.19			0.23		
Esters	Acetic acid, 1-methylpropyl ester	735.45			8.04							
	1-Butanol, 2- methyl-, acetate	735.83				7.51	6.55			6.35	7.30	
	Methoxyacetic acid, heptyl ester	799.91				0.84	0.24					
	Acetic acid, [(phenylmethoxy) imino]-, trimethylsilyl ester	828.88			0.52		0.15					
	Methoxyacetic acid, octyl ester	891.27			0.24		0.16					1.56
	alpha.-Terpinyl acetate	1396.10							11.34	2.43		1.36
	Benzeneacetic acid, 2-tetradecyl ester	1444.95			0.40	0.36	0.95			0.36	0.31	
	4,7-methano-1H-inden-5-ol, 3a,4,5,6,7,7a- hexahydro-, acetate	1471.88										1.66
	Tricyclo[4.2.1.1(2,5)]dec-3-en-9-ol, acetate, stereoisomer	1474.30			1.58	1.40				1.42	1.31	
	Indan-1,3-diol monoacetate	1475.77					1.29					
	9-Octadecenoic acid (Z)-, phenylmethyl ester	1485.77				0.77	1.42				0.28	0.31
	Benzeneacetic acid, 3-tetradecyl ester	1501.54			0.56		0.16			0.25		0.34
	10,13-Octadecadiynoic acid, methyl ester	2016.07			0.21							
	Phthalic acid, butyl undecyl ester	2103.38				0.22						
Acids	4-Methyloctanoic acid	1034.66									1.10	1.15
	Benzeneacetic acid	1372.14			2.13		1.22				0.87	
	phenyl-Propanedioic acid	1397.22			0.85	1.26			0.64	1.31	0.86	1.00
Ketones	Acetoin	732.39					0.68					
	3-hydroxy-2-Butanone	735.64					0.23					
	2-Hexanone	749.56					0.46					
	4-methyl-2-Hexanone	809.57			0.44						0.72	
	5-methoxy-2-Pentanone	814.22				0.63					1.57	
	5-methyl-2-Hexanone	815.30			1.82	1.48	2.14			1.37		0.94
	3,6-dimethyl-Octan-2-one	1031.51			1.94	0.86	0.35			1.16	0.28	
	2-t-Butyl-5-propyl-(1,3) dioxolan-4-one	1168.02			0.26		0.18					
	3,6-dimethyl-Octan-2-one	1228.56					0.18				1.13	
	4-(3,3-Dimethyl-but-1-ynyl)-4-hydroxy-2,6,6-trimethylcyclohex-2-enone	1410.95			0.51	0.47	0.67			0.48		0.42
	5-Acetyl-4,6,6-trimethylcyclohexa-2,4-dienone	1441.23					0.45					
	2,5-bis (1,1-dimethylpropyl)-2,5-Cyclohexadiene-1,4-dione	1489.34			0.44	0.59	0.82			0.61	0.40	0.76
	2,6-di (t-butyl)-4-hydroxy-4-methyl-2,5-cyclohexadien-1-one	1526.85			1.02	0.91					0.85	1.01
	4-(1-Hydroperoxy-2,2-dimethyl-6-methylene-cyclohexyl)-pent-3-en-2-one	1634.58				0.32						
	4,4-Dimethyl-3-(3-methylbut-2-enylidene)octane-2,7-dione	1635.89			0.33		0.33			0.32		
Aldehydes	3-benzyloxy-2-fluoro-4-methoxy-Benzaldehyde	1696.97			0.36	0.27						
Sulpher compounds	Sulfurous acid, decyl 2-pentyl ester	1133.09			0.58	0.67	0.75			0.70	0.71	0.75
Oxygenated compounds	Taurultam	1116.58							0.77			
	Taurolin	1209.44							1.08			
Miscellaneous	Sec-Butyl nitrite	733.60								6.43		6.78
	[[(1-ethenyl-1,5-dimethyl-4-hexenyl)oxy]methyl]-Benzene	1396.65				0.20					0.20	
	1-benzyloxy-2,4-Difluorobenzene	1696.81				0.27						0.40

Based on the results, the dominant compound in the Con_1_ extract was Butylated hydroxytoluene (BHT). BHT is an antioxidant known to be safe for use in foods (35, 36). Since BHT is a phenolic compound, the dominant odor in the extract was phenolic. The Con_1_ extract, which is the first control sample, was prepared to test the bacteria and induce them to produce volatile compounds responsible for flavors.

Butylated hydroxytoluene exhibits strong antioxidant activity. As an antioxidant, it can provide a hydrogen atom for active free radicals, especially due to the activation provided by both the aromatic ring and hydroxyl group (37).

Furthermore, Butylated hydroxytoluene (66.83%) has been identified as the main component produced from defatted soybean meal fermented using *Aspergillus fumigatus F-993* or *A. awamori FB-133* (38).

#### GC/MS analysis of the SLT_1_ extract

After testing the *Bacillus subtilis NRCH123* strain for the production of flavor compounds, it was stimulated with a mixture of the two amino acids L-Lysine and L-Threonine to identify the compounds responsible for the flavors of the food additive. Table 2 presents the compounds produced from the fermentation of soybean meal by *Bacillus subtilis NRCH123* (SLT_1_ extract) to which L-Lysine and L-Threonine were added. The compounds were categorized into three groups: aliphatic compounds, phenolic compounds, and alcohol compounds.

**FIGURE 2:**
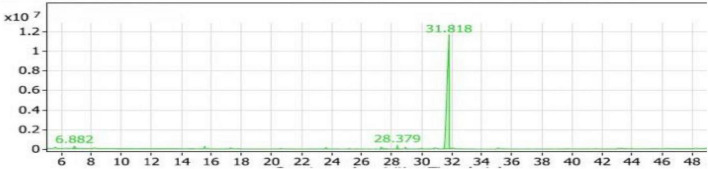
Gas chromatography-mass spectrometry (GC/MS) chart of soybean meal fortified with L-Lysine + L-Threonine fermented by *Bacillus subtilis NRCH123* using solid-state fermentation.

In Table 2, two compounds were identified belonging to the group of aliphatic compounds: 2,4-dimethylheptane and *Trans-*caryophyllene, with a combined percentage of 2.37% of the total compounds. In the group of phenolic compounds, only one compound, 2,6-di-tert-butyl-4-methylphenol (Butylated hydroxytoluene), was identified, representing 96.40% of the compounds with a retention time of 31.818 (Figure 2). The group of alcohols had the lowest percentage (1.23%) and was represented by the compound E-11,13-Tetradecadien-1-ol.

From the results shown in Table 2 and Figure 2, it is evident that the major compound present in the SLT_1_ extract was Butylated hydroxytoluene. Similar to the Con_1_ extract, the dominant odor was phenolic.

#### GC/MS analysis of the SL_1_ extract

The volatile compounds in soybean meal fermented by *Bacillus subtilis NRCH123* enriched with L-Lysine (SL_1_ extract) consisted of 42 compounds belonging to nine groups. Table 2 presents the distribution of these compounds. The largest group of eight compounds belongs to aliphatic compounds, accounting for 39.08% of the total compounds. The highest percentage within the aliphatic compounds was 3-ethyl-3-methyl-pentane, approximately 46.95%, with a retention time of 5.565. Di-sec-butyl ether followed with a percentage of around 42.20%. Another compound in the aliphatic group was 3-ethyl-3-methylheptane, representing approximately 5.99% of the total aliphatic compounds. The only compound in the phenols group was 4,6-di-tert-butyl-m-cresol, accounting for 0.91% of the compounds.

**FIGURE 3:**
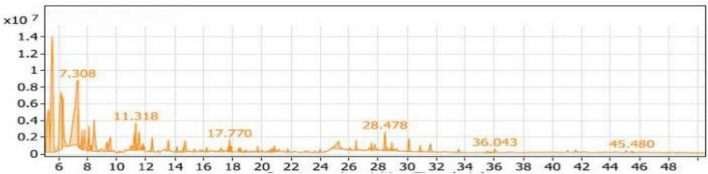
Gas chromatography-mass spectrometry (GC/MS) chart of soybean meal fortified with L-Lysine and fermented by *Bacillus subtilis NRCH123* using solid-state fermentation.

The alcohols group accounted for 37.75% of the compounds, with 19 compounds identified. Among them, Trimethylsilylmethanol had the highest percentage within the alcohols group, approximately 52.19%, with a retention time of 7.308 (Figure 3). It was the most abundant alcohol compound in the SL_1_ extract. The second-highest percentage within the alcohols group was 2-ethynyl-bicyclo\[2.1.1\]hexan-2-ol, with a percentage of 11.47%.

Seven compounds belonged to the esters group, with Acetic acid, 1-methylpropyl ester being the dominant compound, accounting for 69.61% of the total esters (11.55%) in Table 2 and Figure 3. Additionally, three compounds were categorized as acids, representing 2.98% of the compounds, while the ketones group comprised 11 compounds with a percentage of 6.56%. There was one compound from the aldehydes group (0.36%) and another from the sulfur group (0.78%).

Based on the obtained results, the top compounds were Trimethylsilylmethanol, 3-ethyl-3-methyl-pentane, and Acetic acid. However, these compounds, despite having the highest percentages, were not identified as flavoring compounds or off-flavors. Therefore, it is not recommended to use the SL_1_ extract as a food flavoring.

#### GC/MS analysis of the ST_1_ extract

Table 2 presented the nine groups of volatile compounds obtained from the fermentation of soybean meal enriched with L-Threonine by *Bacillus subtilis NRCH123* (ST_1_ extract). The aliphatic compounds group accounted for the highest percentage, 46.86%, and included 11 different compounds. The major compound within this group was 3-ethyl-3-methyl-pentane, representing approximately 41.83% of the total volatile compounds in the ST_1_ extract. It had a retention time of 5.577 and was the most abundant compound in the extract. Di-sec-butyl ether followed in the aliphatic compounds group with a percentage of around 40.55%, while 5,6-dimethyl-decane had the lowest percentage, 0.51%.

**FIGURE 4:**
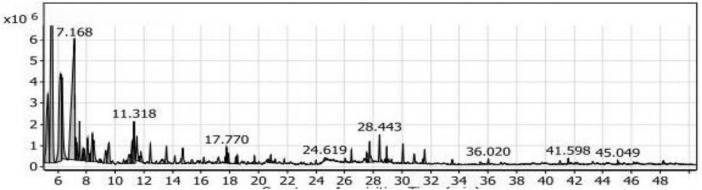
Gas chromatography-mass spectrometry (GC/MS) of the SL_1_ extract of soybean meal fortified with L-Threonine fermented by *Bacillus subtilis NRCH123* using solid-state fermentation.

The phenols group comprised three compounds, accounting for a low percentage of approximately 1.32%. The alcohols group represented 32.52% of the compounds and included 20 compounds. The highest percentage within this group was Trimethylsilylmethanol, approximately 57.07%, with a retention time of 7.168 (Figure 4). It was followed by 2,4-dimethyl-3-pentanol with a percentage of 5.87%. The esters group contained seven compounds, representing 11.10% of the compounds. The dominant compound in this group was 1-butanol, 2- methyl-, acetate, accounting for 67.66% of the esters group (7.51% in the extract).

The acids group accounted for 1.54% of the compounds and consisted of only one compound, phenyl-propanedioic acid. The ketones group comprised seven compounds, representing 5.26% of the compounds. Among them were 5-methyl-2-hexanone and phenyl-propanedioic acid. The aldehydes group had a percentage of 0.27% and included the compound 3-benzyloxy-2-fluoro-4-methoxy-benzaldehyde. The sulfur group contained one compound, sulfurous acid, hexyl 2-pentyl ester, with a percentage of 0.67%. Additionally, there were two compounds in the miscellaneous group, accounting for 0.47% of the compounds.

Based on the obtained results, the highest compounds in the ST_1_ extract were 3-ethyl-3-methyl-pentane and Trimethylsilylmethanol. However, these compounds are not commonly used as flavoring agents, so it is not recommended to use the ST_1_ extract as a food flavor additive.

#### GC/MS analysis of the SY_1_ extract

**FIGURE 5:**
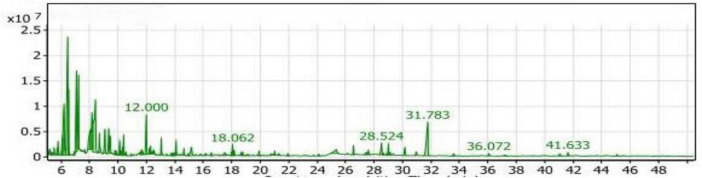
Gas chromatography-mass spectrometry (GC/MS) chart of soybean meal fortified with yeast extract fermented by *Bacillus subtilis NRCH123* using solid-state fermentation.

Table 2 presented a total of eight groups of volatile compounds resulting from the fermentation of yeast extract-fortified soybean meal by *Bacillus subtilis NRCH123.* The SY_1_ extract contained 14 compounds in the aliphatic compounds group, accounting for 41.05% of the total compounds. The main components within this group were 3-ethyl-3-methyl-pentane and di-sec-butyl ether, representing approximately 32.69 and 31.47%, respectively. Four compounds belonging to the phenols group were identified, accounting for 7.14% of the compounds. The highest compound within this group was 2,6-ditert-butyl-4-methylphenol, with a percentage of 77.31% and a retention time of 31.783 (Figure 5).

The alcohols group had the largest number of compounds and the second-highest value after the aliphatic compounds, accounting for 33.19% of the compounds. Trimethylsilylmethanol was the highest-value compound within the alcohols group, representing approximately 46.88% of the alcohols group and 15.56% of the total compounds in the SY_1_ extract. The esters group comprised eight compounds, representing 3.32% of the compounds. One compound in this group was indan-1,3-diol monoacetate, accounting for 1.29% of the total extract compounds with a retention time of 28.524 (Figure 5).

The ketones group had a value of 6.72%, with the major compound being 5-methyl-2-hexanone at a percentage of 31.85%. Acetoin was also a compound within the ketones group, accounting for 10.12% of the group. The sulfur group included only one compound, sulfurous acid, decyl 2-pentyl ester, at a percentage of 0.75%. Sec-butyl nitrite did not fall into any of the previous groups and was placed in the miscellaneous group with a percentage of 6.45%.

Based on the obtained results, the main compounds identified were 3-ethyl-3-methyl-pentane and 5-methyl-2-hexanol. These compounds are not commonly used as flavoring agents. The commonly used compound are present in small percentage, therefore proved to be not effective here. Additionally, the compounds that are commonly used as flavoring agents in foods present in the extract had very small percentages that would not be effective. Therefore, the SY_1_ extract is not recommended as a food flavor additive.

Several types of essential oils and their individual components such as Trimethylsilyl methanol are used as natural antimicrobial compounds to reduce the impact of microbial activities in food products. Mostly, the phenolic components of essential oils also are effectively acting as membrane permeabilizers (39).

Acetoin is a common food flavor additive. This volatile compound widely exists in nature and is primarily responsible for cheese flavor (40). Some microorganisms have the ability to synthesize acetoin using different enzymes and pathways under certain circumstances (41).

Finally, in the five treatments that were carried out on strain *Bacillus subtilis NRCH123*, no extract of flavor compounds appeared that could be added as flavoring agents to foods. In the first and second treatments the dominant compound was Butylated hydroxytoluene, which is one of the antioxidant compounds produced from a natural source, so it is recommended to replace it with industrial antioxidants. It is also recommended to change the conditions and nutrients with the soybean meal during its fermentation with *Bacillus subtilis NRCH123* to stimulate it to produce flavor.

#### GC/MS analysis of the Con_2_ extract

**FIGURE 6:**
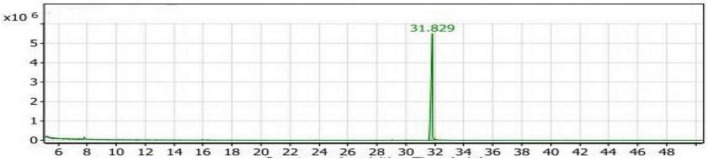
Gas chromatography-mass spectrometry (GC/MS) chart of soybean meal fermented by *Bacillus subtilis NRCZ144* using solid-state fermentation.

The strain *Bacillus subtilis NRCZ144* was tested for producing flavor compounds during fermentation of soybean meal using solid state fermentation, and the control sample was without adding nutrients to the growth medium. Volatile compounds in soybean meal fermented by *Bacillus subtilis NRCZ144* (Con_2_ extract) are shown in Table 2 and in the GC/MS chromatogram in Figure 6. One phenol compound, Butylated hydroxytoluene, appeared with retention time of 31.829.

From these results, the Con_2_ extract is the second control sample, as it contains 100% of the a phenol-list antioxidant compound Butylated hydroxytoluene.

Butylated hydroxytoluene is widely used as an antioxidant food additive, while food-grade Butylated hydroxytoluene is not less than 99% (w/w) pure (42, 43). Phenolic antioxidants such as Butylated hydroxytoluene are used in a wide variety of consumer products including certain foodstuffs better to give examples. 2,6-Di-Tert-Butyl-4-methylphenol is considered generally safe as a food preservative when used at approved concentrations (44).

#### GC/MS analysis of the SLT_2_ extract

**FIGURE 7:**
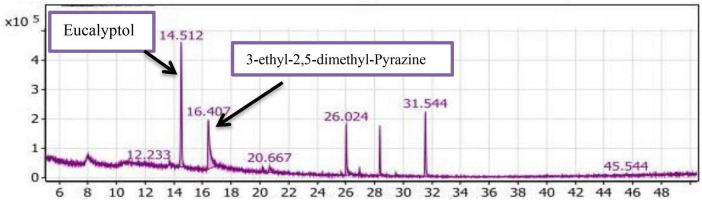
Gas chromatography-mass spectrometry (GC/MS) chart of soybean meal fortified with L-Lysine + L-Threonine fermented by *Bacillus subtilis NRCZ144* using solid-state fermentation.

Table 2 and Figure 7 present the recovered amounts and retention times of six groups of volatile compounds identified in materials extracted from soybean meal fortified with L-Lysine + L-Threonine (SLT_2_ extract) and containing the SSF culture of *Bacillus subtilis NRCZ144.* The aliphatic group compounds accounted for 68.63% of the total, with Eucalyptol being the highest compound, representing 45.53% of the aliphatic group and 31.25% of the entire extract. The second highest compound in the aliphatic group was 3-ethyl-2,5-dimethyl-pyrazine, comprising 38.09% of the aliphatic compounds and 26.14% of the extract. Caryophyllene accounted for 12.02% of the aliphatic compounds, followed by Copaene at approximately 1.75%. Butylated hydroxytoluene, representing the phenolic group, comprised 15.00% of the extract and was the third highest compound overall. Terpineol, representing the alcohol group, constituted 2.55% of the extract and had a retention time of 20.667 (Figure 7).

The esters group was represented by alpha-Terpinyl acetate, accounting for 11.3% of the extract, while Phenyl-Propanedioic acid represented the acids group at 0.64%. Two compounds in the oxygen group, Taurolin and Taurultam, collectively represented 1.85% of the extract (Table 2).

Based on the results in Table 2, it can be concluded that the major compound in the SLT_2_ extract is Eucalyptol, which imparts a strong camphor odor, so the dominant odor in the extract was the strong camphor odor. Eucalyptol biosynthesis is mediated by *bornyl diphosphate synthase* produced by *Bacillus subtilis NRCZ144* during the fermentation of soybean meal. Eucalyptol is a colorless liquid, a natural organic compound, and it is approved by the US Food and Drug Administration (FDA) for use in food preparations to enhance odor and taste (45). In another study, 3-ethyl-2,5-dimethyl-pyrazine was found to contribute significantly to a bean-like aroma (46).

Caryophyllene is a bicyclic sesquiterpene naturally occurring in essential oils from various medicinal and edible plants and is used as a flavoring agent (47). Alpha-terpinyl acetate has therapeutic properties and has been considered as a potential source of new drugs due to its ability to inhibit acetylcholinesterase, butyrylcholinesterase, and reduce oxidative stress and antioxidant capacity, among other effects (48).

Finally, *Bacillus subtilis NRCZ144* is capable of producing strong and palatable flavor compounds that have potential applications in the food, medicine, and cosmetics industries. Eucalyptol, as a major compound, can be isolated from the extract and used alone or in combination with other compounds. The strain also produces a significant percentage of pyrazine, a widely used flavoring compound in the food industry, along with functional compounds.

#### GC/MS analysis of the SL_2_ extract

**FIGURE 8:**
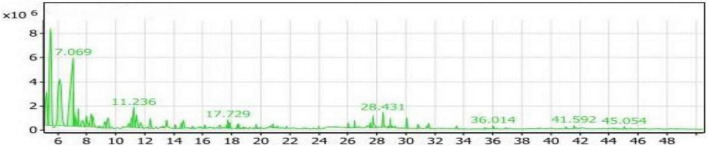
Gas chromatography-mass spectrometry (GC/MS) chart of soybean meal fortified with L-Lysine fermented by *Bacillus subtilis NRCZ144* using solid-state fermentation.

Table 2 and Figure 8 presented the volatile compounds produced from the fermentation of soybean meal after adding L-Lysine by *Bacillus subtilis NRCZ144* (SL_2_ extract). The results indicate that there are 53 volatile compounds categorized into 8 groups. The group with the highest average percentage (48.57%) is the aliphatic compounds group, comprising a total of 15 compounds. The next group is alcohols, consisting of 20 compounds with an average percentage of 33.93%. The diverse group accounts for 6.43% of the compounds, followed by ketones (3.94%) and esters (2.89%). The phenols group represents 2.01% of the compounds, followed by acids (1.31%) and finally the sulfur group (0.70%).

Table 2 also highlights the main compounds in the SL_4_ extract. The highest compound within the aliphatic compounds group is 3-ethyl-3-methyl-pentane, accounting for 41.14% of the group and 19.98% of the entire extract. Trimethylsilylmethanol, belonging to the alcohols group, is the next prominent compound, comprising 56.62% of the group and 19.21% of all compounds. It has a retention time of 7.069, as indicated in the chromatogram (Figure 8). Di-sec-butyl ether holds the third position with 34.16% of the aliphatic compounds. Another noteworthy compound in the extract is Sulfurous acid, hexyl 2-pentyl ester, which possesses antibacterial, antiviral, and antifungal properties, and potentially affects cancer cells (49).

As the case into Table 2, the compounds with the highest percentages are non-flavor compounds and cannot be added as flavoring agents to foods.

#### GC/MS analysis of the ST_4_ extract

**FIGURE 9:**
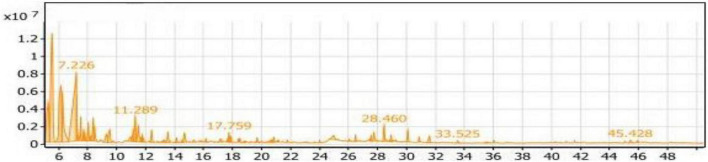
Gas chromatography-mass spectrometry (GC/MS) chart of soybean meal fortified with L-Threonine fermented by *Bacillus subtilis NRCZ144* using solid-state fermentation.

Table 2 and Figure 9 showed the volatile compounds produced from the fermentation of soybean meal containing the amino acid L-Threonine by *Bacillus subtilis NRCZ144* (ST_4_ extract). Table 2 demonstrates 56 volatile compounds categorized into nine groups. The groups with the highest percentages are aliphatic compounds (46.18%) and alcohols (33.19%), while esters make up 10.41% of the compounds. The groups with lower percentages are ketones (5.38%) and acids (2.83%). The groups with rates less than one include phenols (0.84%), sulfur (0.71%), aldehydes (0.26%), and miscellaneous compounds (0.20%).

Within the aliphatic compounds group, Table 2 suggests the presence of 18 compounds. The major compounds in this group are 3-ethyl-3-methyl-pentane, Di-sec-butyl ether, and 1-(1-methylpropoxy)-Butane, with percentages of 39.24, 25.53, and 15.54%, respectively.

The alcoholic group comprises 18 volatile compounds, and the major compound in the extract is Trimethylsilylmethanol, accounting for 18.64% of the group and 56.16% of the aliphatic compounds. It has a retention time of 7.226, as indicated in the chromatogram (Figure 9). The main compound in the esters group was 1-Butanol, 2- methyl-, acetate, which constitutes 70.12% of the group.

As the case in Table 2, the compounds with the highest percentages in the ST_2_ extract are not flavored and should not be added to foods as flavoring agents.

#### GC/MS analysis of the SY_2_ extract

**FIGURE 10:**
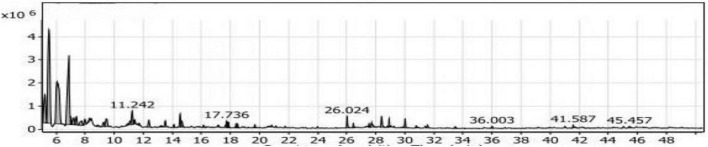
Gas chromatography-mass spectrometry (GC/MS) chart of soybean meal fortified with yeast extract fermented by *Bacillus subtilis NRCZ144* using solid-state fermentation.

The volatile compounds produced from the fermentation of fortified soybean meal with yeast extract by *Bacillus subtilis NRCZ144* (SY_2_ extract) were listed in Table 2 and some of them are shown in the chromatogram in Figure 10. The main groups in SY_2_ extract were aliphatic (54.1%) and alcoholic (26.3%), each containing 14 volatile compounds. The compound 3-ethyl-3-methyl-pentane occupied almost half of the aliphatic group at a rate of 46.95%, followed by Di-sec-butyl ether (33.83%), including Eucalyptol was a low rate of about 3.46%.

The same Table showed Trimethylsilylmethanol, it was the third largest compound in the extract and the first in the alcohol group, with a rate of 17.21 and 65.43%, respectively. The secondary groups present in the extract are the esters (5.23%) and contain five compounds, the ketones (2.19%) and have three compounds, the acids (2.15%) and contain two of the compounds. As for the phenols (1.13%), it had three compounds as well, and the sulfur group (0.75%) had only one compound. There were also two compounds that did not belong to any of the previous groups, so they were included in the miscellaneous group (7.18%).

From the results mentioned above and demonstrated in Table 2, the compounds with the largest percentage in SY_2_ extract are not flavored and should not be added to foods as flavoring additives.

Ultimately, Strain 4 is unable to produce flavor compounds when fermenting unfortified soybean meal and fortified with L-Lysine alone or L-Threonine alone as well as yeast extract, although it produces flavor with soybean meal fortified with a mixture of L-Lysine and L-Threonine under the same conditions. Thus, it is recommended to change the conditions of the medium, as well as use other growth promoters and different amino acids to stimulate the strain to produce flavor compounds that can be added to food.

Therefore, the SY_2_ extract was chosen to conduct some tests to find cellular toxicity and antioxidant properties, given that it contains flavor compounds that can be added to food products.

Finally, the solid-state fermentation of soybean meal using the *Bacillus subtilis NRCZ144* strain and fortified with a combination of 2.5% (w/w) L-Lysine and 2.5% (w/w) L-Threonine (SLT_2_ extract) has demonstrated the ability to produce robust and pleasant flavor compounds. These compounds have potential applications in the fields of food, medicine, and cosmetics. Notably, Eucalyptol is one of the prominent compounds identified in the extract, and it can be isolated and utilized either alone or in combination with other compounds.

Furthermore, the strain exhibits a remarkable capability to produce a high percentage of pyrazine, a flavoring compound widely utilized in the food industry. Additionally, the strain also has the potential to produce functional compounds with beneficial properties. Therefore, this study selected the extract (SLT_2_), to investigate the biological potential of this particular extract.

### Cytotoxicity assay of SLT_2_ extract

**FIGURE 11:**
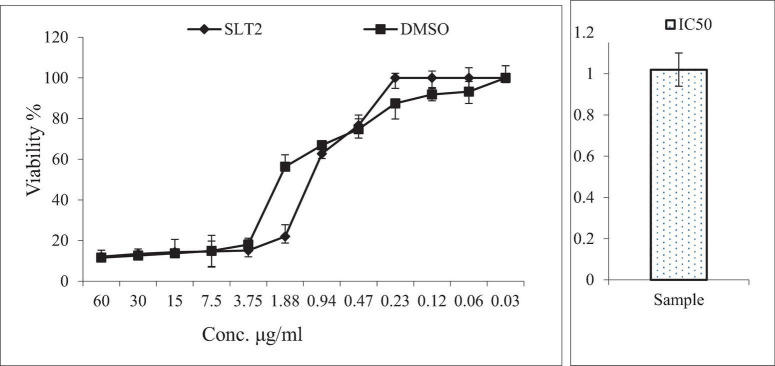
Cytotoxicity assay of SLT_4_ extract concentrations compared to DMSO and IC_50_ against Vero cell lines.

Cytotoxicity is the toxicity brought on by chemotherapeutic drugs acting on live cells. The cytotoxicity of SLT_2_ extract was evaluated using the African green monkey (Vero) cell lines through the MTT assay, comparing it with dimethyl sulfoxide (DMSO). The concentrations of LT_2_ extract and the examined DMSO ranged from 60 μg/mL to 0.03 g/mL, as shown in Figure 11.

Figure 11 presented the data on the toxic effects of SLT_2_ extract on normal cells, aiming to assess its cytotoxicity compared to a positive control sample. The MTT assay study revealed that SLT_2_ extract exhibited some cytotoxicity toward Vero cell lines, with an IC50 (concentration causing 50% inhibition) approaching 1.02 μg/ml.

Based on the data obtained from Figure 11, it can be observed that at the highest tested concentration of SLT_2_ extract (60 μg/ml), there was a decrease in cell activity equivalent to that of the positive control sample, with the percentage of live cells being 12.06% and 11.57% for SLT_2_ extract and the positive control sample, respectively. Additionally, at concentrations of 30, 15, and 7.50 μg/ml, the percentage of live cells was less than 50%. However, at a concentration of 0.23 μg/ml, no cell death was recorded.

These results demonstrate that the compounds present in the SLT_2_ extract do not exhibit toxicity to normal cells at low concentrations. However, at higher concentrations of the same extract, some toxicity is observed. Regarding Eucalyptol, itis a colorless liquid and a natural organic compound commonly found in eucalyptus essential oil. Eucalyptol is widely used in pharmaceutical industry and food preparations as a flavoring agent (50).

It is generally recognized as safe (GRAS) by regulatory agencies, such as the U.S. Food and Drug Administration (FDA) and the European Food Safety Authority (EFSA). These agencies have evaluated Eucalyptol’s safety profile and determined that it does not pose significant negative effects on animals when used in appropriate amounts (51).

IC_50_: The half maximal inhibitory concentration.

### Anticancer activity of SLT_2_ extract

An irregular growth of cells or tissues in body is called cancer (52). There is significant global interest in research on cancer prevention, and it is claimed that adopting a healthy lifestyle and using natural agents can help prevent certain types of cancer. Natural substances, including dietary phytochemicals and polyphenols, have been identified as having chemopreventive properties. Several natural substances are currently being studied in clinical trials to assess their safety and effectiveness in preventing or treating human cancer (53).

**FIGURE 12:**
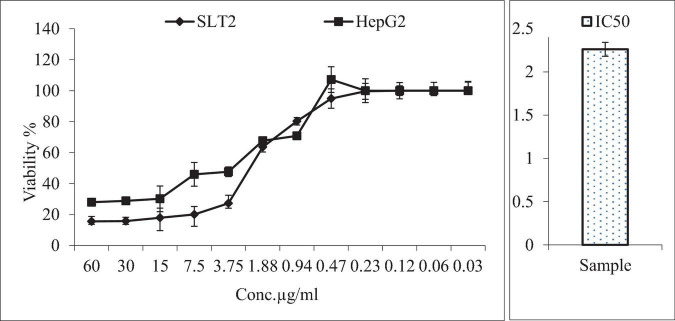
Anticancer activity of SLT_2_ extract concentrations compared with Cisplatin and IC_50_ against human hepatocellular carcinoma cell line.

Figure 12 presented the results of an MTT assay, which assessed the inhibition and viability effects of different concentrations of SLT_2_ extract and Cisplatin on the human liver cancer cell line (HepG2). Twelve concentrations ranging from 0.03 to 60 μg/mL of SLT_2_ extract were prepared to study its inhibitory effect on the HepG2 cell line. Both Cisplatin and SLT_2_ extract did not exhibit cytotoxicity at concentrations below 0.94 μM, as expected. However, as the concentrations of the positive control sample and SLT_2_ extract increased, the cell viability decreased, as shown in Figure 12.

Furthermore, Figure 12 indicated that at the maximum concentration of 60 μg/mL, the cell death resistance was low, resulting in an inhibition of 84.4 and 72% for the SLT_4_ extract and Cisplatin, respectively. The lowest inhibitory concentration of the SLT_4_ extract and the positive control sample on the cell lines was 0.94 μg/mL. The inhibitory concentration causing toxic effects of the SLT_2_ extract, expressed as IC50, was reported as 2.26 μg/mL.

The anticancer potential of Eucalyptol, an active compound found in eucalyptus essential oil, was evaluated on HepG2 liver cancer cell lines. Cell viability and proliferation were determined using the MTT assay. HepG2 cell lines were treated with various concentrations of Eucalyptol (0, 5, 10, 25, 50, and 100 μg/mL) for 24 h. The results showed that proliferation in HepG2 cancer cells was potentially inhibited in a dose-dependent manner. The IC50 value for Eucalyptol was determined to be 11.3 μg/mL. The sub-lethal dose was considered to be between 10 and 25 μg/mL, which resulted in survival rates of 52.2 and 36.2% in HepG2 cells. It was observed that Eucalyptol insignificantly inhibited cell growth up to a concentration of 25 μg/ml (54).

Eucalyptol is an active compound of eucalyptus essential oil and was reported to have many medical attributes including cytotoxic effect on cancer cells (55). Additionally, other studies have investigated the effects of eucalyptol on different cancer types, such as breast cancer, lung cancer, and skin cancer (56). These studies have reported varying degrees of cytotoxic effects, including inhibition of cell proliferation, induction of apoptosis (programmed cell death), and disruption of cancer cell signaling pathways (57, 58).

IC_50_: The half maximal inhibitory concentration.

### Antiviral activity of SLT_2_ extract

Foodborne viral infections pose a significant threat to public health and have a detrimental impact on both industrialized and developing nations’ economies. Enteric viruses are the primary cause of outbreaks and illnesses associated with foodborne pathogens. Egypt is listed by the World Health Organization (WHO) as having intermediate to high endemicity for several enteric viruses. This is evidenced by the high prevalence rates of various enteric viral diseases, including Hepatitis A, among the Egyptian population (59).

**FIGURE 13:**
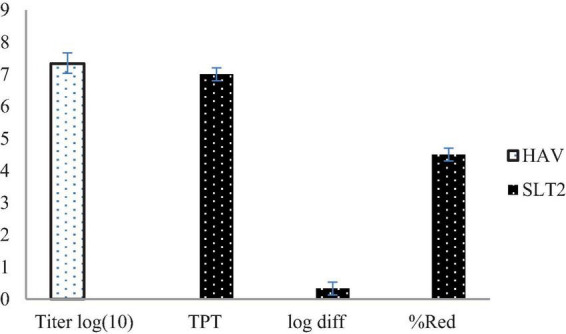
Antiviral assay of SLT_2_ extract against Hepatitis A virus using cell culture.

SLT_2_ extract has been reported to reduce the infectivity titers of Hepatitis A virus (HAV) after treatment at a safe concentration of the extract, as previously determined on Vero cells, as shown in Figure 13. The results indicate that the extract demonstrated a decrease in viral infection titer by 0.33 log _(10)_ / 0.1 ml following treatment.

According to the data presented in Figure 13, the virus titer before treatment with the SLT_4_ extract was 7.33 log _(10)_ / 0.1 ml, which decreased to 7 log _(10)_ / 0.1 ml after treatment with the extract. The recorded data also revealed that the SLT_2_ extract exhibited a low potential against the same virus, with a reduction rate of 4.5%.

Several studies have suggested potential antiviral activity of eucalyptol also known as 1,8-cineole. Influenza Virus: Influenza viruses are respiratory viruses that cause seasonal flu outbreaks, (60). Herpes Simplex Virus (HSV): HSV is a common virus that causes oral and genital herpes infections (61). Respiratory Syncytial Virus (RSV): RSV is a respiratory virus that primarily affects young children and can cause severe respiratory infections (62).

TPT: titer post-treatment. %Red: reduction percentage.

### Antioxidant activity of SLT_2_ extract

**FIGURE 14:**
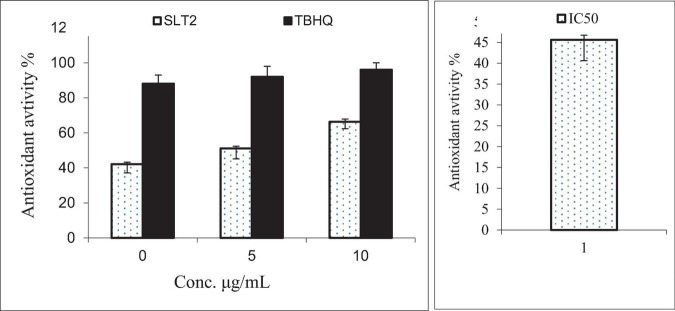
Antioxidant activity of SLT_2_ extract compared with Tert-butyl hydroquinone (TBHQ) and IC_50_ against DPPH assay.

When the body is exposed to various environmental stresses, it generates free radicals, which are highly reactive and unstable molecules. These free radicals can cause oxidative stress and damage to cells. Cellular antioxidants play a crucial role in defending against the harmful effects of these free radicals (63). The scavenging activity of free radicals by the 1,1-diphenyl-2-picrylhydrazyl radical (DPPH) method is an antioxidant assay based on electron transfer (64). The data presented in Figure 14 indicates that as the concentration of SLT_2_ extract increased, its ability to scavenge free radicals (DPPH) also increased. At a concentration of 100 μg/mL, the extract demonstrated an effect comparable to TBHQ (a reference antioxidant compound), with a scavenging activity of 96%. The strongest efficacy of the SLT_2_ extract was observed at reducing DPPH radicals by 72.04%. However, at a concentration of 25 μg/mL, the extract exhibited a lower antioxidant activity of 45.4%, while TBHQ showed 88% activity at the same concentration.

Furthermore, in the same figure, at a concentration of 50 μg/mL, the SLT_2_ extract displayed a scavenging activity of 56.7%, while TBHQ showed a value of 92%. The IC50 (effective concentration value) is a parameter used to assess the data obtained from the DPPH method. It represents the substrate concentration that leads to a 50% reduction in DPPH activity. In the case of the SLT_2_ extract, the IC50 value for scavenging activity was determined to be 35.23 μg/mL.

Since the SLT_2_ extract contains Eucalyptol, a monoterpene compound (65), it demonstrated strong antioxidant activity. Monoterpenes are a class of organic compounds commonly found in essential oils derived from various plants, including citrus fruits, conifers, and aromatic herbs. Many monoterpenes have been studied for their antioxidant activity due to their chemical structure and ability to scavenge free radicals, Hydrogen Donation, Metal Chelation, Lipid Peroxidation Inhibition, Anti-Inflammatory Effects and Other Mechanisms: such as enhancing the activity of antioxidant enzymes, reducing the production of ROS, and modulating cellular signaling pathways involved in oxidative stress (66). Eucalyptol has shown potential as an antioxidant in various studies, suggesting its ability to scavenge free radicals, mitigate oxidative stress, Lipid Peroxidation Inhibition, Enzyme Stimulation, Anti-Inflammatory Effects and Neuroprotective Potential (67).

TBHQ: Tert-butyl hydroquinone, standard synthetic antioxidant IC_50_: The half maximal inhibitory concentration.

## Antimicrobial activity of SLT_4_ and BLY_2_ extracts

### Antibacterial activity

#### Inhibition zone diameter

**FIGURE 15:**
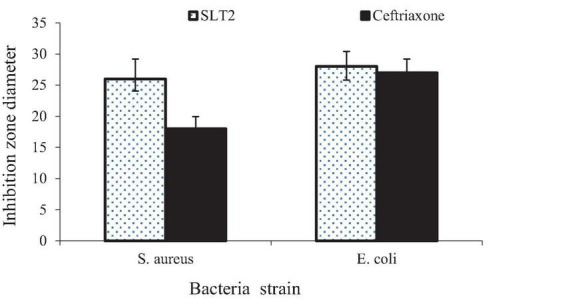
Inhibition zone diameter of SLT_2_ extract compared to Ceftriaxone against bacterial species.

The agar diffusion method was used to measure the inhibitory zone diameters, as shown in Figure 15. The results indicate that the SLT_2_ extract exhibited effectiveness against two tested microbial strains. The inhibition zone diameter was 26 mm for the Gram-positive bacteria *S. aureus* and 28 mm for the Gram-negative bacteria *E. coli.*

The data presented in Figure 15 reveals that the antibacterial activity of the SLT_4_ extract was more potent against E. coli, followed by *S. aureus.* When compared to the standard commercial antibacterial Ceftriaxone (positive control sample), the SLT_4_ extract showed larger inhibition zones, measuring 27.2 mm and 18.7 mm for *E. coli* and *S. aureus*, respectively.

Modern antimicrobial compounds, especially those effective against pathogenic microorganisms, can be derived from essential oils. Essential oils typically exhibit antibacterial activity through multiple mechanisms of action at the cellular level. These mechanisms include irreversible damage to the bacterial membrane, resulting in the loss of cytoplasm, energy substrates (such as glucose and ATP), bacterial lysis, leakage of ions, and ultimately, bacterial death. Another potential mechanism of action is the inhibition of proteases, leading to the coagulation of cell contents (68, 69). Several studies have suggested the potential antibacterial activity of Eucalyptol, a compound derived from essential oils. It has demonstrated broad-spectrum activity, effectively inhibiting the growth of both Gram-positive and Gram-negative bacteria. One of the mechanisms by which Eucalyptol exerts its antibacterial effects is through the disruption of bacterial cell membranes, compromising their integrity and function. Additionally, Eucalyptol has been shown to inhibit the activity of certain bacterial enzymes, further compromising bacterial viability. It also exhibits the ability to reduce the formation of biofilms, which are protective communities of bacteria encased in a matrix. Furthermore, Eucalyptol has shown promising synergistic effects when used in combination with antibiotics, enhancing the overall antibacterial activity. These findings highlight the potential of Eucalyptol as an antibacterial agent and its possible role in combating bacterial infections, although further research is still needed to explore its clinical applications and optimize its use (70).

#### Minimum inhibitory concentration

**FIGURE 16:**
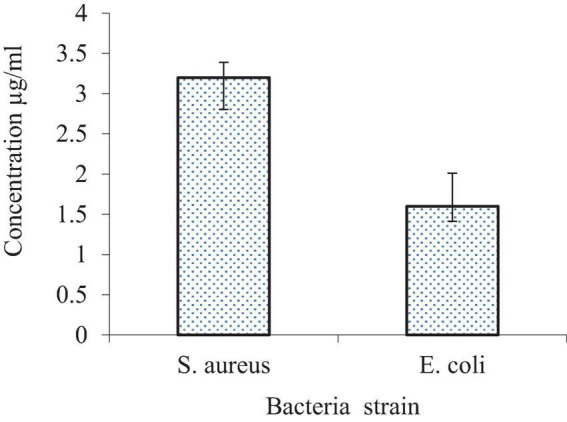
Minimum inhibitory concentration (MIC) of SLT_4_ extract against bacterial species.

The minimum inhibitory concentration (MIC) is a measure used to determine the lowest dosage required to inhibit the growth of a bacterial organism. In this case, the MIC values against the tested bacterial strains were determined, and the results are presented in Figure 16. Prior to the interaction study, the SLT2 extract underwent a microbroth assay to determine the MIC values for *S. aureus* and *E. coli*. Figure 16 displays the lowest MIC values, indicating a high antimicrobial susceptibility of the SLT4 extract. The MIC values were found to be 3.125 μg/mL for *S. aureus* and 1.65 μg/mL for *E. coli.*

Eventually, microbial extracts exhibit strong antibacterial activity at very low concentrations compared to plant extracts containing the same compounds. This is because microbial extracts have higher concentrations of active compounds, resulting in a stronger effect.

### Antifungal activity

#### Inhibition zone diameter

**FIGURE 17:**
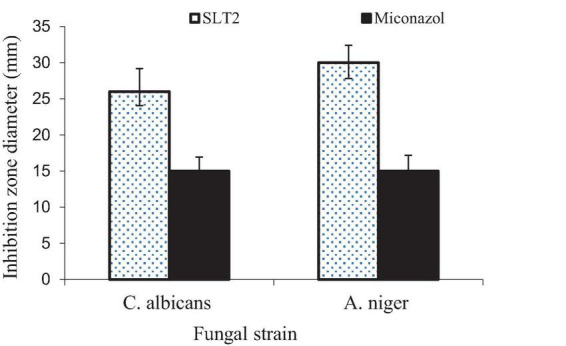
Inhibition zone diameter of SLT_2_ extract compared to Miconazole against fungal species.

The data presented in Figure 17 demonstrates the strong antifungal activity of the SLT_4_ extract against *C. albicans* and *A. niger*, with inhibition zone sizes of 28 and 26 mm, respectively. The results indicate that the antifungal activity of the extract was more effective against *C. albicans* and *A. niger* compared to the standard commercial antifungal Miconazole (positive control sample), which exhibited antifungal activity of 15.2 and 15.8 mm, respectively.

Eucalyptol, also known as 1,8-cineole, has been identified as the primary component in the isolated essential oils derived from *Achillea setacea* and *Achillea teretifolia*. The antimicrobial properties of these essential oils were individually assessed against various microorganisms, including *Candida albicans* (71). The minimum inhibitory concentration (MIC) values ranged from 0.28 to 2.25 mg/mL, indicating the effectiveness of Eucalyptol as a potent antimicrobial agent (72). Notably, Eucalyptol is one of the main components of Listerine, and it has shown activity against bacterial and fungal strains, particularly *Candida albicans* (73). The key points regarding the antifungal activity of Eucalyptol are broad-spectrum activity, disruption of fungal membranes, inhibition of fungal enzymes, anti-biofilm activity and combination therapy (40).

### Minimum fungicidal concentration

**FIGURE 18:**
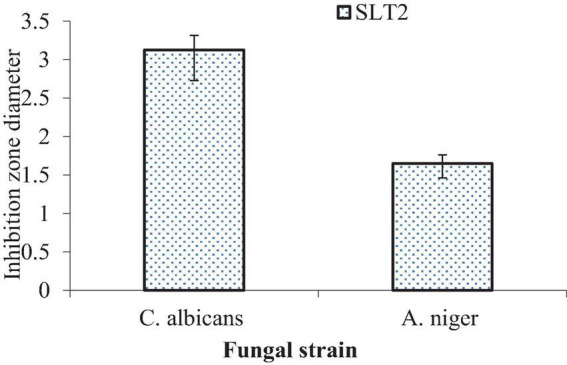
Minimum fungicidal concentration (MFC) of SLT_2_ extract against fungal species.

Results of the MFC values of SLT_2_ extract against potentially pathogenic fungi are illustrated in Figure 18. The data present the lowest MFC value of (3.125 μg/ml) and (1.65 μg/ml) for *C. albicans* and *A. niger*, respectively, which gave very high antifungal susceptibility of SLT_2_ extract.

In a prospective study, it is proposed to evaluate the antifungal activity of SLT_2_ extract on several other fungal strains that cause food spoilage and produce mycotoxins.

## Sensory evaluation of SLT_2_ extract

Sensory evaluation measures individuals’ response to stimuli (74). Sensory evaluation of flavor produced by fermentation of soybean meal by *Bacillus subtilis NRCZ144*, odor detection after 3 days in culture supplemented with amino acids (L-Lysine and L-Threonine). The perceived aroma was described as having a camphor-like flavor. The results of the GC/MS analysis also confirmed the results of the sensory olfactory evaluation of the camphor-like flavor.

**FIGURE 19:**
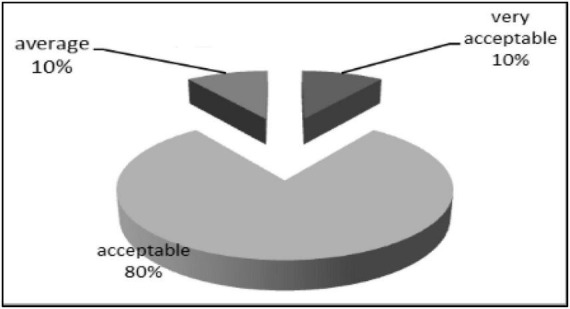
Acceptance score of SLT_2_ extract.

The data presented in Figure 19 showed the degree of acceptance of Eucalyptol that was extracted from the fermentation environments as a percentage of arbitration by the arbitrators. The data indicated that 10% of the arbitrators gave SLT_4_ extract a very acceptable degree, while 80% was acceptable and 10% gave the sample a medium degree. While not record any value in unacceptable or very unacceptable.

Finally, it is recommended to use the Eucalyptol compound, which is responsible for the camphor flavor, as a natural flavoring in food and beverages.

## Conclusion

The *Bacillus subtilis NRCZ144* strain is capable of fermenting soybean meal fortified with L-Lysine and L-Threonine (SLT_2_ extract) and producing acceptable sensory and non-toxic flavor compounds, and has anticancer, antioxidant, antiviral, and antifungal properties. The SLT_2_ extract derived from *Bacillus subtilis NRCZ144* (B2)-fermented soybean meal, fortified with L-Lysine and L-Threonine, exhibits a rich flavor profile, significant antioxidant potential (72.04% at a concentration of 100 μg/mL), anticancer activity (promising anticancer activity against the human liver cancer cell line HepG2, with an IC50 value of 2.26 μg/mL.), cytotoxicity (an IC50 value of 1.02 μg/mL, indicating its ability to induce cell death in a concentration-dependent manner), as well as antibacterial and antifungal properties even at low concentrations. It was also concluded that the microbially produced flavors have wide sensory acceptance. The findings highlight the potential of essential oil-derived compounds, such as Eucalyptol, as effective antimicrobial agents and flavor enhancers. Further research and development in this area could lead to the production of natural and safe alternatives to conventional antimicrobial and flavoring agents. Overall, this study contributes to our understanding of the biological and sensory properties of the SLT_2_ extract and highlights the potential applications of Eucalyptol in various industries, including pharmaceuticals, food, and beverages. Further studies care planned to explore the feasibility of utilizing these flavor compounds in commercial applications. However, further research is needed to fully understand the underlying mechanisms of flavor compounds extraction and evaluate flavor compounds safety and efficacy in different settings.

## Data availability statement

The original contributions presented in the study are included in the article/supplementary material, further inquiries can be directed to the corresponding authors.

## Author contributions

MH: Data curation, Validation, Writing – review and editing. AA-A: Conceptualization, Formal analysis, Methodology, Writing – original draft. MR: Conceptualization, Project administration, Supervision, Visualization, Writing – original draft. MM: Methodology, Writing – review and editing. RS: Data curation, Software, Validation, Writing – original draft. FS: Formal analysis, Software, Validation, Writing – original draft. AZ: Writing – review and editing. TE-M: Validation, Visualization, Writing – review and editing.
